# Case for diagnosis. Ulcerated lesions: a diagnostic challenge in Behçet's syndrome^☆^^☆☆^

**DOI:** 10.1016/j.abd.2021.01.002

**Published:** 2021-05-24

**Authors:** Lissiê Lunardi Sbroglio, Karin Milleni de Araújo, Bianca Passos Leite dos Santos, Alexandre Carlos Gripp

**Affiliations:** Department of Dermatology, Hospital Universitário Pedro Ernesto, Universidade do Estado do Rio de Janeiro, Rio de Janeiro, RJ, Brazil

**Keywords:** Behçet syndrome, Delayed diagnosis, Diagnosis, differential, Signs and symptoms

## Abstract

This study reports the clinical case of a 42-year-old patient with ulcerated lesions who was followed up by general practitioners with the diagnosis of recurrent cellulitis. However, when referred to the Dermatology division a diagnosis of Behçet's syndrome was established based on clinical criteria. Although there are defined clinical criteria for this syndrome, sometimes its diagnosis can be challenging, due to lack of knowledge of the disease and extremely heterogeneous clinical phenotype. The authors highlight the potential difficulties in establishing the diagnosis considering the multiple clinical findings during the investigation process, contributing to the risk of increased morbidity and mortality.

## Case report

**Figure 1:**
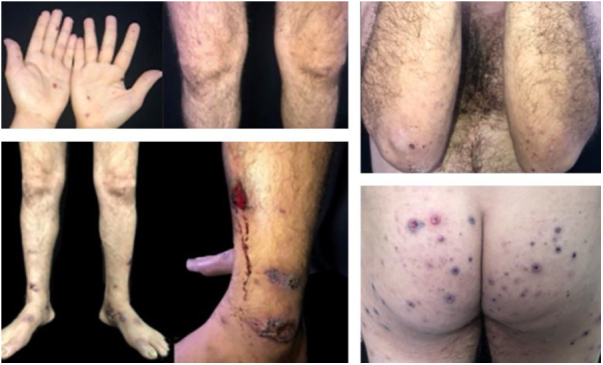
Purpuric lesions on the hands; papulopustular, purpuric, and ulceronecrotic lesions on the lower limbs, and ulceronecrotic lesions on the gluteal region and elbow.

**Figure 2:**
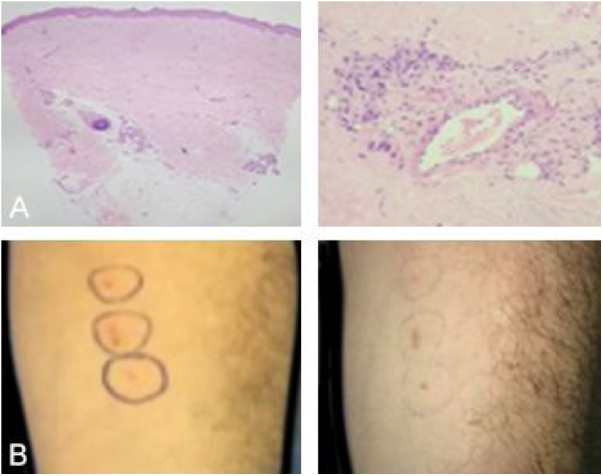
(A), (Left) Anatomopathological analysis showing diffuse cicatricial fibrosis and vascular proliferation (Hematoxylin & eosin, ×40). (Right) Anatomopathological analysis showing perivascular inflammation with a predominance of neutrophils (Hematoxylin & eosin, ×200). (B), (Left) Pathergy test with positive reading (right).

**Figure 3:**
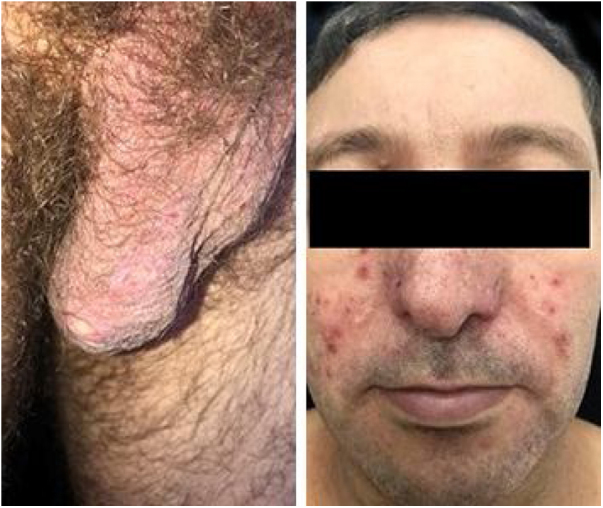
Scrotal ulcers and acne-like lesions on the malar regions.

This is a case report of a male patient, 42 years old, with ulcerated lesions on the lower limbs for 12 months. He had been treated in the primary health care services as a case of recurrent cellulitis. Due to therapeutic failure, after cycles of antibiotics and low doses of corticosteroids, he was referred for evaluation at the authors’ Dermatology Unit. The initial clinical examination revealed papulopustular, purpuric and ulcerative necrotic lesions predominantly located on the lower limbs (Fig. 1). The diagnostic hypotheses were Behçet's syndrome (BS), granulomatosis with polyangiitis, eosinophilic granulomatosis with polyangiitis, and endocarditis. Complementary exams (autoantibody screening, investigation for infectious diseases, echocardiography, electroneuromyography of the lower limbs, chest and sinuses computed tomography and fundoscopy) showed no alterations. The skin biopsy was non specific (Fig. 2) and the pathergy test was positive (Fig. 2). The HLA-B typing test showed the presence of allele 51. During the follow-up, he had acne-like lesions on the face and painful ulcers on the scrotum, with no lesions in the oral mucosa (Fig. 3).

## What is your diagnosis?

a) Granulomatosis with polyangiitis
b) Endocarditis
c) Behçet's syndrome
d) eosinophilic granulomatosis with polyangiitis

## Discussion

**Figure 4:**
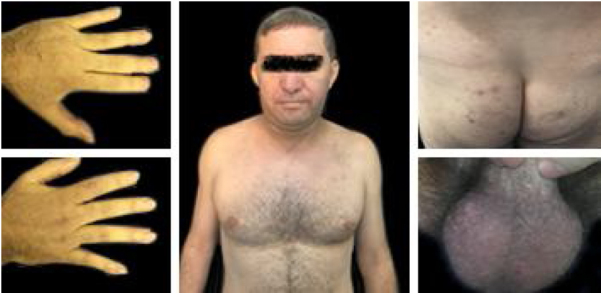
Patient with progressive improvement of the condition after starting drug treatment (July 2019).

Based on the most recent international criteria, the diagnosis of BS was established.^1^ Treatment with colchicine 1.5 mg/day associated with prednisone 0.5 mg/kg/day was started, with disease improvement (Fig. 4). The patient is undergoing follow-up by a multidisciplinary team, with no systemic involvement to date. BS is a multisystemic vasculitis with acute inflammatory outbreaks that affect vessels of any caliber.^1^, ^2^ The multifactorial etiopathogenesis suggests environmental agents (differences in the manifestation pattern in endemic and non-endemic areas), complex genetic factors, mainly associated with the HLA-B51 – MHC class I allele, which is important in neutrophil activation.^3^

The initial and most common characteristics are recurrent oral and genital mucosal ulcerations, which are commonly painful and multiple, with well-defined edges, resolving with punched out scars. Pustular, purpuric, ulcerated lesions, erythema nodosum-like, and migratory thrombophlebitis have been described.^4^ The concept of BS phenotyping is used, with four main phenotypes: skin-mucosal involvement, skin, mucosal and joint involvement, vascular disease, and ocular involvement.^1^ There is no clinical finding or complementary examination which is pathognomonic.^5^ The most recent international diagnostic criteria do not include oral ulceration as mandatory.^6^

Misdiagnoses have been associated with insufficient collection of semiological data and the tendency that some professionals have towards a greater appreciation of technology (laboratory and imaging tests). In this case, no specific complementary exam contributed to the diagnosis, since it is entirely based on clinical criteria, with the exception of the pathergy test.

The treatment aims at symptom remission, not being curative.^5^ Topical agents (corticosteroids, calcineurin inhibitors and antibiotics) are used for mucocutaneous lesions; for extensive/refractory lesions or those associated with musculoskeletal conditions, colchicine, systemic corticosteroids, non-steroidal anti-inflammatory drugs, and dapsone are used; for eye symptoms, azathioprine, cyclosporine, mycophenolate mofetil, thalidomide, and methotrexate are used and for systemic involvement, pulse therapy with corticosteroids and cyclophosphamide and anti-TNF-alpha are the treatment of choice. The following treatments are under study: phosphodiesterase-4 inhibitor, interleukin-1 receptor antagonist, IL-6 and IL-12/23 inhibitors.^7^, ^8^

In the case reported here, despite the exuberance of the cutaneous lesions, there were no lesions on the oral mucosa, which may have influenced the delay in the diagnosis. Considering BS in the presence of ulcerated lesions in the lower limbs, especially if associated with genital and/or oral lesions, is crucial for specific conduct to be adopted, reducing unnecessary treatments and the negative impact on the patients’ quality of life.

## Financial support

None declared.

## Authors’ contributions

Lissiê Lunardi Sbroglio: Design and planning of the study; drafting and editing of the manuscript; critical review of the literature.

Karin Milleni de Araújo: Critical review of the literature; critical review of the manuscript.

Bianca Passos Leite dos Santos: Critical review of the literature; intellectual participation in propaedeutic and/or therapeutic conduct of the studied case.

Alexandre Carlos Gripp: Approval of the final version of the manuscript; critical review of the manuscript.

## Conflicts of interest

None declared.
